# Delegation to artificial agents fosters prosocial behaviors in the collective risk dilemma

**DOI:** 10.1038/s41598-022-11518-9

**Published:** 2022-05-19

**Authors:** Elias Fernández Domingos, Inês Terrucha, Rémi Suchon, Jelena Grujić, Juan C. Burguillo, Francisco C. Santos, Tom Lenaerts

**Affiliations:** 1 Machine Learning Group, Computer Science Department, Université Libre de Bruxelles, 1050 Brussels, Belgium; 2 Artificial Intelligence Lab, Computer Science Department, Vrije Universiteit Brussel, 1050 Brussels, Belgium; 3 IDLab, Ghent University - imec, B-9052 Ghent, Belgium; 4 ETHICS - EA 7446, Université Catholique de Lille, Maison des Chercheurs, 59000 Lille, France; 5 atlanTTic Research Center, E.E. Telecom., Universidade de Vigo, 36310 Vigo, Spain; 6 INESC-ID and Instituto Superior Técnico, Universidade de Lisboa, IST-Taguspark, 2744-016 Porto Salvo, Portugal; 7 Center for Human-Compatible AI, UC Berkeley, Berkeley, CA 94702 USA; 8 FARI Institute, Université Libre de Bruxelles-Vrije Universiteit Brussel, Brussels, 1050 Belgium

**Keywords:** Human behaviour, Social behaviour

## Abstract

Home assistant chat-bots, self-driving cars, drones or automated negotiation systems are some of the several examples of autonomous (artificial) agents that have pervaded our society. These agents enable the automation of multiple tasks, saving time and (human) effort. However, their presence in social settings raises the need for a better understanding of their effect on social interactions and how they may be used to enhance cooperation towards the public good, instead of hindering it. To this end, we present an experimental study of human delegation to autonomous agents and hybrid human-agent interactions centered on a non-linear public goods dilemma with uncertain returns in which participants face a collective risk. Our aim is to understand experimentally whether the presence of autonomous agents has a positive or negative impact on social behaviour, equality and cooperation in such a dilemma. Our results show that cooperation and group success increases when participants delegate their actions to an artificial agent that plays on their behalf. Yet, this positive effect is less pronounced when humans interact in hybrid human-agent groups, where we mostly observe that humans in successful hybrid groups make higher contributions earlier in the game. Also, we show that participants wrongly believe that artificial agents will contribute less to the collective effort. In general, our results suggest that delegation to autonomous agents has the potential to work as commitment devices, which prevent both the temptation to deviate to an alternate (less collectively good) course of action, as well as limiting responses based on betrayal aversion.

## Introduction

Intelligent autonomous agents are already present at many levels in our daily lives. Examples can be found in a diverse range of domains: autonomous vehicles^1,2^, fraud detection^3–5^, personal home assistants^6,7^, call centers^8^, drones^9^, recommendation systems^10^ or educational support systems^11,12^. These intelligent agents facilitate many of our individual needs while also influencing different aspects of our social lives. Yet, there are still many unknowns about how relying on artificially intelligent (AI) systems alters our choices and decision-making, and, particularly, the balance between selfish and cooperative behaviors. Thus, their presence in social contexts raises questions about the role they play on our capacity to achieve common goals and, ultimately, what impact these artificial intelligent (AI) systems have on social welfare.

In this manuscript, we address some of these questions experimentally, using a game-theoretical perspective. Our behavioural economic experiments investigate how human decision-making is affected when people (1) delegate their actions to autonomous agents, whether provided by third parties or coded by the participants themselves, and (2) interact in hybrid human-agent groups where participants do not know who are humans or agents. In the first context, our experiment also examines if having the capacity to “code” or customize the agent with one’s own norms affects the observed behavior. While this provides additional insight into the effect of delegation on decision-making, it also provides insight into how people believe the agents should behave when they are acting on their behalf.

In all experiments—delegation, customization of the agent, and hybrid interactions—we adopt the Collective-Risk Dilemma (CRD)^13–19^, a threshold public goods game^20–23^ with uncertain and delayed returns. The CRD has been used to abstract social decision-making problems such as climate action, abuse of antibiotics or collective hunting, where participants aim to avoid a future loss, rather than to seek a benefit. It is a type of public goods game, but with a delayed reward and potential for serious adverse effects when the objective is not achieved. Whereas research on this kind of public goods games has been focused on humans, there is no reason to assume that these tasks cannot be addressed by autonomous agents, where they act as proxies for their owners. The owners will be able to exert some control about what these agents will do in their name, but once decided, they are out of the loop.

Delegation to AI is happening all around us. Examples range from self-driving cars to road safety, where humans delegate not only the responsibility of driving the car to an AI, but also a series of moral decisions^24,25^. Another example is the increasing presence of conversational (chat) bots in social media, which help broadcasting information and recommending content, but may have an influencing effect which has the potential to both promote more cooperative behaviors as well as spread corrupted (unethical) ones^25,26^. In relation to the CRD, the additional assumptions are that the individual AI are involved in a group effort for which rewards are only assigned at the end, when the outcome of the collective effort is finally due. An example could be delegating the sharing of joint energy sources (e.g. solar panels and connected batteries) in an apartment building, where each tenant’s AI needs to ensure her users’ needs, while not draining the common battery.

Thus, the CRD nicely captures the dilemmas emerging from the examples above, but also abstracts many other important social situations that may at some point involve an AI proxy. These may include the use of finite energy resources, where humans may delegate decisions about how to manage their house appliances to an AI, or, more generally to environmental governance^13,27^. Here, several elements play a key role, such as communication^15,28^, wealth and income inequality^15,16^, sanctions and positive incentives^18,29,30^, among many other factors.

In the CRD, a group of human participants is confronted with a choice: contribute sufficiently (cooperate) over several rounds to achieve a collective target, ensuring that the group benefit is achieved, or contribute insufficiently (defect) and assume that others will make the contributions to reach the goal, and thus, aim to maximise one’s personal gains. Concretely, in our setup of the CRD, participants interact in groups of 6, where each member is required to contribute either 0, 2 or 4 Experimental Monetary Units (EMUs) in every round to a public account out of their private endowment (40 EMUs). The experiment consists of 10 rounds and, in the end, if the sum of the total contributions of all players is higher or equal than a collective target of 120 EMUs, then all players will keep the remainder of their endowment. Otherwise, all players lose their remaining endowment with a probability of $90\%$, which represents the collective risk of the dilemma and thus gain nothing. In earlier experiments, Milinski et al.^13^ evaluated how different risk probabilities affect human decision-making in the CRD. They found that most groups facing high risks (p=0.9) succeed while most groups facing lower risk fail. Fernández Domingos et al.^19^ repeated the treatment with $90\%$ of risk of failure^13^, finding similar results. We use this treatment as a control for the experiment presented in this manuscript.

Here, we contrast three treatments with this *only humans* control. In the first treatment we explore what happens when the CRD is not played directly by humans but instead by artificial agents they select from a small set of possibilities to play on their behalf. These agents either display unconditional (always plays the same action in any give round) or conditional (adapt their behavior to the rest of the group) behaviors, and have been designed following the experimental results of the CRD and model presented in^19^. This experiment examined, on the one hand, which agents are selected and, on the other hand, whether groups of agents are more or less successful than groups of *humans*. In a second treatment, all participants get the same template-agent and are asked to *customize* (rudimentary programming) the agent so it follows an algorithm that represents their beliefs/preferences on how the game should be played. At the same time, we elicited whether participants prefer to play themselves or through agents. In a third treatment, referred by *nudge treatment*, we examine what happens when the group consists of a combination of humans and agents, where the latter are selected from the successful groups in the *customize* treatment, hypothesizing that, given their prior success in solving the CRD, they may stimulate coordination within the group: is there any difference in the success rate compared to a CRD with only humans or only agents? Again the answer depends on the effect of different agent behavior combinations in the group, as different choices are possible. Here we asked what would happen in the “best” case, thus, selecting the agents programmed by participants from successful groups from the previously mentioned customisation experiment (see details below). Moreover, we evaluate whether participants think agents contributed more or less than human participants.

The remainder of the paper is organised as follows. In the next section we give an overview of the experimental and theoretical literature about delegation and hybrid interactions with autonomous agents. Afterwards, we present and discuss our experimental results and later we summarise our final conclusions. In the final “Methods” section and the Supplementary Information (SI), we provide additional details on our experimental design and protocols.

## Background

The *fundamental* question of how the presence of autonomous agents may affect human social interactions has been explored from sociological, strategical and psychological perspectives^31–38^. In behavioural economics, where strategic interactions between individuals are analysed, March surveys more than 90 experimental studies of interactions between humans and artificial intelligence^32^, and finds that humans tend to act in a more self-interested or selfish manner when computer players are present, and, often, they are able to exploit such players. Also, Cohn et al.^39^ show that individuals tend to cheat about three times more when interacting with agents instead of another human, and that dishonest individuals prefer to interact with agents. As artificial intelligence (AI) evolves, the opposite situation may also occur, i.e., intelligent agents may exploit humans. Camerer^33^ argues that behavioural experiments are necessary to understand and predict how this may happen.

The literature on delegated interactions mostly focuses on human-human delegation. These type of interactions are often studied through the principal-agent relationship framework^40–42^, where principals delegate their actions/tasks to agents. Hamman, Lowenstein and Weber^41^ show, through an experiment in which participants engage in a modified dictator game, that principals tend to choose agents that will donate the least. This results in a decrease in donations compared to the control treatment, in which participants directly choose how much to donate. The authors find that principals use delegation to deflect the blame, or responsibility, for their actions by delegating them to the agents. Nevertheless, Bartling and Fischbacher^42^ find that when punishment is present, the rate of fair outcomes with delegation is very similar to a situation without delegation. Moreover, the frequency of participants willing to delegate significantly increases in the presence of punishment, since delegation allows the punishment to be shifted to the agent, who, in exchange, also tends to choose fairer outcomes. Furthermore, Gawn and Innes^43^ find that introducing the option to delegate in a binary dictator game reduces generosity. This is because dictators, who would be generous when choosing directly, use delegation as a curtain from which to hide, reducing their moral costs for not being generous. They tend to shift the blame for their decisions to the delegates while preserving their chance to maximize their private payoffs. These results heavily emphasize the possible negative impact of delegation in a collective system, but indicate that such impact may be reduced by imposing punishment mechanisms.

On the other hand, Corazzini, Cotton and Reggiani^44^ show that delegation in the setting of threshold public goods games may increase the chances of reaching positive outcomes. They show that delegation in this context facilitates coordination and increases contributions to the public good. These results are in line with other works in the literature which indicate that this type of electoral delegation promotes positive outcomes^45,46^. These contrasting observations suggest that the effect of delegation may be dependent on the context, and be particularly well suited for coordination problems such as threshold public good games.

Fewer works have examined the impact of delegation to autonomous artificial agents. Initial results by de Melo et al.^35^ have shown experimentally that delegating to programmable autonomous machines, also known in the literature as peer-designed agents^47,48^, changes the way in which participants approach a social dilemma and promotes the evaluation of long-term consequences of choices, which in their case increases cooperation. In this sense, delegation to autonomous agents also acts as a commitment device, motivating long-term prosocial behavior and preventing coordination failures due to the temptation to deviate from ones’ initial plan. Overall, this promotes socially better outcomes. In another experimental study, the same author^49^ finds that participants behave more fairly when acting through machines in comparison to when human participants directly interact with others. They also hypothesise that when participants program the agents they tend to rely on social norms, which could explain the increase in fairness. At the same time, the authors report the same results when participants were requested to indicate their actions for each possible game scenario before the game started instead of being told that they had to program an agent.

Additionally, introducing a delegation system to machines programmable/customizable by users to represent their interests in social settings may have several benefits. They can be used to reduce costs in the design and testing of mechanisms that involve human decision-makers, such as automated negotiations and mechanism evaluation^50,51^, agent-based simulations^48^ or testing experimental hypothesis in game-theoretic scenarios. At the same time, those applications assume that the peer-designed agents truly represent their designer strategy/behavior. This, however, may not always be true and may depend on the context of the decision-making scenario. For instance, Grosz et al.^52^, Elmalech et al.^53^ and Manistersky, E.^54^ find that, in a negotiation (or trading) setting, participants design agents closer to rational game-theoretic models which, in this context, means agents which are more selfish, instead of more fair or cooperative agents. This contrasts with human behavior observed in direct interactions^55,56^ and the increased fairness discussed by Melo et al.^35^. Nevertheless, other authors find that programmed agents can increase the ability of participants to coordinate when negotiating^57^.

As previously mentioned, humans have been reported to act more selfishly when interacting in hybrid groups than in groups composed of humans only^32^. However, de Melo et al.^58^ showed experimentally that, by adding visual, emotional and cultural cues to the agents, cooperation with agents can be achieved. Concretely, the authors find that the level of cooperation between humans and agents using emotional cues (e.g., sadness, joy) is very similar to that observed in human-human interactions, and higher than in the absence of such emotional cues. Emotional cues can also alter how humans assign reputations to others, suggesting a new spectrum of emotion-based social norms of relevance when interacting in hybrid groups of humans and machines^59^. This research also hints that trust is a very important factor in human-agent interactions. In this line, Andras et al.^31^ suggests that a framework of trust between humans and autonomous agents must be developed. Moreover, it also prompts further research on how such trust currently affects hybrid strategic interactions. Han et al.^60^ argues that trust-based strategies can be very effective in human-agent interactions, particularly when there is a lack of transparency about the agents’ strategies.

In general, experimental results appear to be dependent on the context of the strategic interaction, generating two competing hypotheses: (1) interaction with or through autonomous agents affects social preferences in such a way that it promotes more selfish behaviours and thus decreases cooperation; (2) interacting through and with autonomous agents may eliminate or reduce negative effects of emotions such as revenge or *fear of betrayal*^61–65^, favoring pro-social behaviours and thus cooperation.

## Results

**Figure 1:**
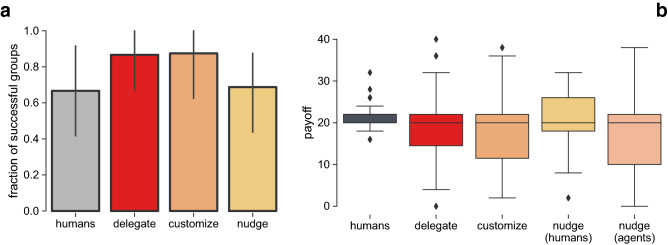
Group achievement and average payoff. Panel (**a**) compares the fraction of successful groups observed in the *humans* control treatment (black: $n=12$, $66.7\%$) to the results of the *delegate* (red: $n=15$), *customize* (orange: $n=8$) and *nudge* (yellow: $n=16$) treatments. There is an increase in groups that achieve the target on the *delegate* ($87\%$) and *customize* ($87.5\%$) treatments, however this trend is not maintained in the *nudge* treatment ($68.8\%$). Panel (**b**) shows the distribution of payoffs obtained by participants of the four treatments. The results indicate that the variance of payoffs increases in all treatments with delegation of hybrid interactions.

In Fig. 1a we show the fraction of successful groups for each of our treatments. In the treatment with only *humans* (gray bar), $66.7\%$ ($n=12$) of the groups successfully achieved the collective target, despite the high level of risk ($p=0.9$). In a previous CRD experiment, Milinski et al.^13^ observe an even lower result (fraction of successful results$=0.5$, $n=10$). This outcome does not appear to change in the *nudge* (yellow bar) treatment ($68.8\%, n=16$), yet, for the *delegate* (red bar) and *customize* (orange bar) treatments success increases to $87\%$ ($n=15$) and $87.5\%$ ($n=8$) respectively. Yet, Fig. 1b shows that the variance over payoffs increases in all of the treatments in comparison to the *humans* control treatment, which can be indicative of an increment in payoff inequality among participants.

**Figure 2:**
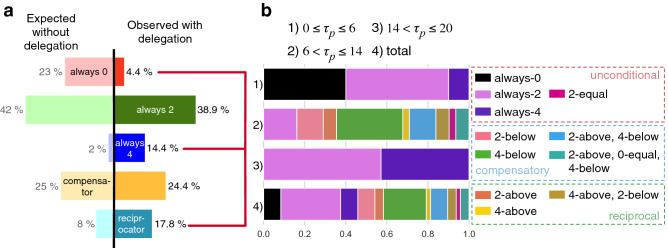
Delegation bias. Panel (**a**) shows the distribution of agent behaviors chosen by the participants (right of the black line) in the *delegate* treatment ($n=90$) in comparison to the expected values predicted by the Fernández Domingos et al. model^19^ (left of the black line). The observed values indicate that there is a bias toward behaviors that are beneficial for the success of the group. Distribution of agent configurations in the customize treatment. Panel (**b**) displays the distribution of configurations for the three levels of personal threshold and in total ($n=10$, $n=31$, $n=7$ and $n=48$, respectively). Always-0 only appears for participants that configured low values of $\tau _{p}$, while participants that selected high values of $\tau _{p}$ choose always-4 with a similar frequency than always-2. The biggest variety of configurations is observed for intermediate levels of $\tau _{p}$ where participants give preference to conditional behaviours and select with higher frequency a 4-below (compensatory) configuration. In general, the most frequent behaviours are always-2 and 4-below.

To better understand this increase in group success with delegation, in Fig. 2 we show the distribution of choices of agent behavior in the *delegate* and *customize* treatments. Figure 2a compares the distribution of agent behaviors chosen by participants (right of the black line) in the *delegate* treatment ($n=90$) with the expected distribution predicted by an evolutionary game-theoretical model^19^ (left of the black line). The behaviors which are included in this model are based on observations of individual behavior from data of the *humans* control treatment, and were used to design the 5 agents of this *delegation* treatment. We observe an increase in the always-4 and reciprocal agents while there is a decrease in always-0.

Making such comparison is more complicated in the case of the customize treatment, due to the high number of possible agent configurations. These customizable agents (see details in “Methods” section) can adapt their contributions in relation to the rest of the group. At each round, starting from round 2, the agents will compare their personal threshold ($\tau _{p}$) to the sum of contributions of the other members of the group ($a_{-i}(t-1)$) in the previous round, and can make different contributions (0, 2 or 4) when $\tau _{p}$ is bigger, smaller or equal to $a_{-i}(t-1)$. For example, if $\tau _{p}=10$ and $a_{-i}(t-1)=6$, a *2-above* agent, will contribute 0 EMUs, while a *2-below* agent will contribute 2. In Supplementary Fig. S1a we can see that most participants have selected values for the personal threshold parameter, $\tau _p$, between 6 and 14. For this reason, in order to simplify the analysis of the agents’ behaviors in this treatment, we divide the agents into three groups: those that have a low ($0\le \tau _p \le 6$) or high ($14 < \tau _p \le 20$) personal threshold, and those that selected intermediary values ($6 < \tau _p \le 14$). In this way, we consider that only agents with $6 < \tau _p \le 14$ can display conditional behaviors, since in the other two cases, the chances for the condition to activate are very scarce. Figure 2b displays the distribution of configurations for the three levels of personal threshold and in total ($n=10$, $n=31$, $n=7$ and $n=48$, respectively). Always-0 only appears for participants that configured low values of $\tau _{p}$, while participants that selected high values of $\tau _{p}$ choose always-4 with a similar frequency as always-2. The biggest variety of configurations is observed for $6 < \tau _p \le 14$ where most participants selected conditional behaviours and selected with higher frequency a 4-below (i.e, contribute 4 EMUs if the contributions of the other member of the groups are below $\tau _{p}$) configuration (see Fig. S2 in the SI for additional details about how the behaviors were grouped). We consider this behavior to be compensatory, since the agent contributes if the other members of the group are not contributing enough. Other similar compensatory behaviors appear in the population with less frequency: 2-below, (2-above, 4-below) and (2-above, 0-equal, 4-below). The last two behaviors, although of a compensatory nature, can also be considered cooperative, since most of the time they will make non-zero contributions. We also observe some reciprocal behaviors: 2-above, 4-above and (4-above, 2-below). In general, the most frequent behaviours are always-2 ($31.3\%$) and the compensatory behaviors ($41\%$), while reciprocal behaviors decrease to $10.4\%$ and always-0 increases to $8.3\%$.

**Figure 3:**
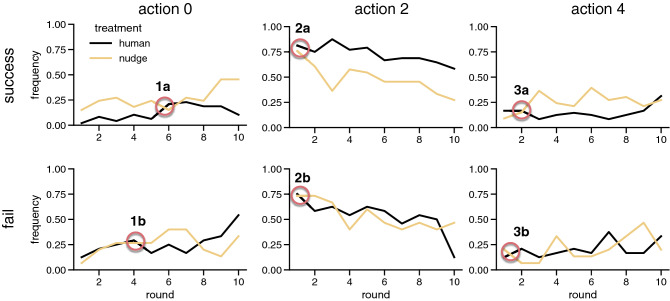
Frequency of each possible action over the 10 experimental rounds for successful and failed groups. The subplots display the frequency of the contributions (0, 2 or 4) made by human participants over the 10 rounds of the game separated for successful and failed groups. The subplots in the second column show that in both the *humans* (black lines) and *nudge* (yellow lines) treatments, independent of whether the group was successful or not in reaching the target, participants are inclined towards contributing 2 EMUs in the first round (points 2a and 2b). Also, in both treatments, there is a decreasing trend in the frequency of action 2. The first column indicates that there is a slight increase in the frequency of action 0 in the *nudge* treatment with respect to the *human* control (points 1a and 1b). Finally, the third column shows that, in successful groups, participants in the *nudge* treatment contribute 4 EMUs more frequently than in the *humans* treatment after round 2 (point 3a). This is not true for the unsuccessful groups (point 3b) where we observe that the frequency of this action oscillates.

In the hybrid human-agent treatment, as shown in Fig. 1, we did not observe the same increase in group success as in the delegate and customize treatments. Nevertheless, we do observe some important differences between this treatment and the *humans* control. Figure 3 shows the frequency of each possible contribution (i.e., 0, 2 or 4) made by human participants over the 10 rounds of the game separated between successful and failed groups. We observe that in both the *humans* (black lines) and *nudge* (yellow lines) treatments, independent of whether the group was successful or not in reaching the target, participants are inclined towards contributing 2 EMUs in the first round (points 2a and 2b). This also appears to be the main first action selected by participants in the *customize treatment* (see Supplementary Fig. S1b). Also, in both treatments, there is a decreasing trend in the frequency of action 2. On the contrary, in successful groups, participants in the *nudge* treatment contribute 4 EMUs more frequently than in the *humans* treatment after round 2 (point 3a). This is not true for the unsuccessful groups where we observe that the frequency of this action oscillates (point 3b). Surprisingly, the frequency of action 0 increases in the successful groups of the *nudge* treatment with respect to the *humans* treatment. Moreover, the main difference between successful and failed groups appears to occur within the 4th and 6th rounds, in which failed groups increase their frequency of contributing 0 EMUs (point 1b), while successful groups slightly decrease it (point 1a) and only increase it again after round 8. Additionally, in Supplementary Fig. S3 we show that the contributions of agents and humans in successful groups are synchronized from the start of the game, while unsuccessful groups are desynchronised by one round. Moreover, Supplementary Fig. S4 indicates that hybrid groups contribute in a more polarized manner than in the *humans* treatment, with more players contributing either more or less than half of their endowment, which is considered the fair contribution. Yet, they also tend to make higher earlier contributions, which can help avoid coordination problems in the later stages of the game.

**Figure 4:**
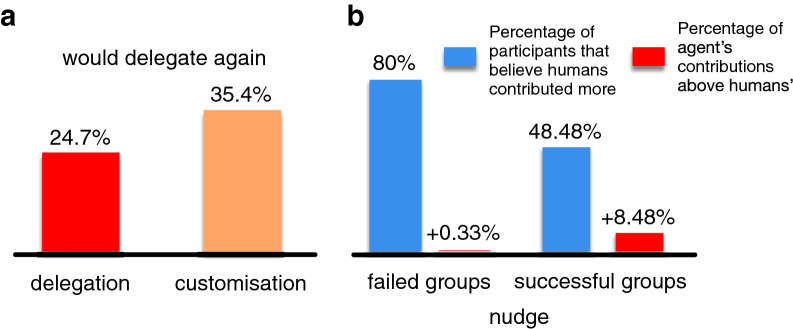
Willingness to delegate and trust in the artificial agents. Panel (**a**) shows the percentage of participants that would choose to delegate ($n=89$) or customize ($n=48$) an agent to play in their place if they were given the option to participate again in the experiment by themselves or to choose an agent. These results show that most participants would rather participate in the experiment by themselves, despite the higher rates of group success in comparison to the experiments with only humans. Nevertheless, there is an increase in participants that would delegate again in the *customize* experiment. This could indicate that allowing the customisation of artificial agents in CRD systems could be a potential relevant design feature to increase their penetration across users. Panel (**b**) shows the percentage of participants who consider that humans had made the greatest contribution effort during the *nudge* treatment, compared to the average contribution difference of agents with respect to humans in a group. These results show that while $80\%$ of the human players from failed groups ($n=15$) believed humans contributed more during the experiment, artificial agents had marginally contributed more ($+0.33\%$). Such a result indicates a strong perception bias that attaches guilt of failure to the artificial agents. This perception decreases in participants from successful groups, where only $48.48\%$ believe humans had put most of the effort ($n=33$), yet the extra effort made by the agents is only and $8.48\%$ higher than that of the human players.

Finally, in treatments *delegate* and *customize* we asked participants in a post-experiment questionnaire whether they would prefer to delegate/customize or make their own contributions decisions if they would play the game again. Figure 4a shows the percentage of participants that would choose to delegate ($n=89$) or customize ($n=48$) again when given the choice. These results show that most participants prefer to act in the experiment themselves. Nevertheless, a higher proportion of participants would delegate again in the *customize* experiment than in the *delegate* treatment. Additionally, in the *nudge* treatment, we asked participants in the post-experiment questionnaire whether they thought humans or agents made the highest contributions during the experiment. Figure 4b shows the percentage of participants who believe that humans made the greatest contribution effort and the actual difference in the average contribution of agents and humans. These results show that while $80\%$ of the human players from failed groups ($n=15$ – 5 groups) believed humans contributed more during the experiment, artificial agents actually marginally contributed more ($+0.33\%$). This perception decreases in participants from successful groups, where only $48.48\%$ believe humans had put most of the effort ($n=33$ – 11 groups), yet the extra effort made by the agents is $8.48\%$ higher than that of the human players.

## Discussion

We have studied experimentally how delegated, and hybrid interactions affect human behaviour in a high risk CRD. Participants in treatments that require interaction through autonomous agents (delegation) tend to choose/customize more cooperative agents, avoiding extremely greedy or free-riding strategies. This has a direct impact on the collective success (or group achievement), which increases (delegate: 86.67%; customize: 87.50%) with respect to the control treatment where humans play the CRD (humans only - 66.67%). However, this trend does not continue in the nudge treatment (68.75%), which suggests that our initial hypothesis about the possibility of our agents nudging higher levels of cooperation was incorrect. Moreover, in all the treatments (delegation, customize and nudge) there is an increase in the variance of the payoffs with respect to the *human* control, which results in a rise in payoff inequality. This effect highlights the importance of balancing the end objective of increasing group success with improving the wealth distribution in our experimental population and should be further investigated in future research.

A lot of evidence from behavioral economics shows that negative emotions may get in the way of cooperation between humans. For instance, humans fear betrayal which can reduce their propensity to contribute^66^ and react strongly when they feel betrayed (negative reciprocity see cite). These negative impacts of emotions on cooperation are reduced when humans act through autonomous machines^34,35,49^, because they nudge participants to make long-term plans - rather than thinking on the short-term - and are commitment devices forcing humans to stick to their plan. These several factors play an important role in the observed increase of cooperation.

At the end of the delegation experiments, participants were asked whether, if given the option to participate in the experiment again, and if delegation was optional, they would choose to delegate to an artificial agent, or to play the game themselves. The results indicate that most of the participants in both treatments (delegate and customize) would prefer to not delegate. These choices suggest a reluctance to give away agency, and a preference for retaining control over the actions at each round. Yet, in the customize treatment, there was an increment in the fraction of participants that would prefer to delegate again, suggesting that the ability to customize/configure the agent increases participants’ trust that the agent will carry out their goals, which is a relevant feature when designing delegation systems. Therefore, future research should explore combining delegation and customisation to achieve both cooperation and user satisfaction.

The group success in the *nudge* treatment is very close to that observed in the *humans* control. However, although most participants continue to contribute 2 EMUs in the first round, in successful groups, we see an increase in the frequency of contributions of 4 EMUs (see Fig. 3). This results in an increase of the level of contributions in the first half of the game as shown in Supplementary Fig. S4b, which is important to avoid possible coordination errors in the last rounds of the game. Moreover, Supplementary Fig. S3 shows that the contributions of agents and humans in successful groups are synchronized from the start of the experiment, which highlights the importance of the presence of agents in those groups. Thus, our experiment shows that these hybrid interactions have the potential to nudge positive collective behavior change in the CRD, especially since coordinated action is required from early stages of the game.

It is also worth noticing that, although the presence of agents in the *nudge* treatment is able to promote more contributions of 4EMUs in the human participants of successful groups, in both the *humans* and *nudge* treatment, there is a decreasing trend in the frequency of 2 EMUs contributions over time. This indicates that most players want to initially signal their cooperative intentions, but later do not follow this commitment, and react accordingly to the others’ actions. As explained before, this may be due to emotions (e.g., fear of betrayal) and can be solved through delegation to artificial agents. Notwithstanding, it may also be due to the nature of the strategies players follow, which as shown by Fernández Domingos et al.^19^, mostly consist on waiting to see what others are doing and compensate in the last rounds of the game. These compensatory strategies can be mostly considered selfish: Players initially signal their intention to cooperate, however are not willing to put any more effort than necessary to reach the collective goal, and are happy to let others contribute in their place. For this reason, we believe future work should focus on the promotion of reciprocal responses instead of compensatory ones.

Finally, the results of the post-experiment questionnaire, in which we ask participants whether they thought humans made more contributions to the public pot than agents, or the other way around (see Fig. 4b) show that most participants believe artificial agents contribute less than human participants, even though this is not true. This suggests that there exists a negative perception towards artificial agents, i.e. they are associated with a higher degree of selfishness than humans. On the other hand, and however we would require another experiment to prove this hypothesis, a hybrid group seems to focus human attention on the agents, indirectly producing a similar reduction in betrayal aversion as observed in the delegation treatments. Moreover, since most participants were not able to identify which members of the group were humans or artificial agents (see Supplementary Figs. S5 and S6), this effect does not appear to be related to a particular agent behavior, but rather by the fact that the group itself contains artificial agents.

## Conclusions

Previous research has shown that people, when delegating to other humans, tend to choose those who act more selfishly^63,67^. In contrast, observations throughout this experiment suggested that people prefer to delegate their decisions to agents that benefit the collective, thus increasing cooperation. This conclusion is also supported by previous experiments with autonomous agents^35^ and 2-player dilemmas. Overall, our results indicate that delegation as we define may act as a long-term commitment device. Yet, subjects still prefer to retain control of their actions, diminishing the potential positive impact of artificial proxies. Our experiment shows that this limitation can be partially overcome if participants have the chance of customizing the agent chosen to play on their behalf. In this case, we observe an increase in the fraction of participants that were willing to delegate again. One should also take into account that, when interacting with these customized agents, subjects in hybrid groups tended to assign the responsibility of failure to the autonomous agents and not to other human participants. Further investigation should address the interplay of social expectations towards autonomous agents, both when delegating and in hybrid interactions, such that participants can embed their preferences and social norms into their delegates. Work along these lines is in progress.

## Methods

To explore experimentally the questions exposed in the introduction, we designed three experimental treatments that test how the presence of autonomous agents influence participant behaviour. These treatments are compared to a control experiment in which only humans participate. In all treatments, participants play a CRD in a group of 6 members for 10 rounds with a collective target of $T=120$ EMUs. Each participant starts with a private endowment of $E=40$ EMUs and will lose their remaining endowment at the end of the game with probability $p=0.9$ if the joint contributions of the group do not reach the target.

### Control treatment with only humans

In the control experiment, human participants from each session are randomly sampled in groups of 6. All their decisions are anonymous, i.e., neither the other participants nor the experimenters know the identity of the participant making the decisions. The experiment is held in a laboratory and participants interact with each other through a computer interface. In each round, each participant is able to observe their own contribution and the contributions of the other participants in the previous round, allowing them to adopt conditional decisions. At the end of the experiment, participants are shown the result of the experiment. In case their group did not reach the target, the computer generates a random number between 0 and 1 and if this number is smaller than .9 all participants lose the remaining of their endowment. The generated number is shown to participants. Finally, they have to complete a short survey that elicits their motives during the experiment. The full description of the results of this treatment are presented in Fernández Domingos et al.^19^.

### *Delegate* treatment (T1)

**Figure 5:**
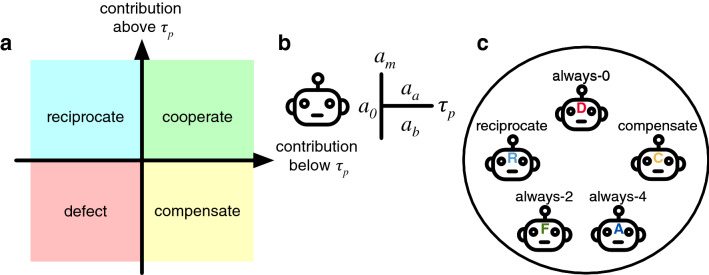
Representation of agent behaviour. Panel (**a**) classifies the behaviour of agents in relation to how much they contribute in response to a stimuli that is above or below a certain personal threshold $\tau _p$. When an agent contributes the same positive amount for stimuli below or above the threshold, we say that it cooperates. When it contributes more for values below (above) the threshold, we say that the agent compensates (reciprocates). Finally, when the agent does not contribute, we say that it defects. Panel (**b**) shows a general structure for the description of the agent’s behaviour. An agent has 5 parameters that consist on an initial contribution ($a_{0}$), a personal threshold for the external stimuli ($\tau _{p}$), and the contributions to be made when the external stimuli is above ($a_{a}$), equal ($a_{m}$), or below ($a_{b}$) $\tau _{p}$. Panel c) represents the pool of agents that can be selected in the *delegate* treatment. Participants may select between cooperative agents (e.g., always-2, always-4), conditional agents (e.g., reciprocal, compensator) and defecting agents (e.g., always-0), however, these names are not shared with them, and agents are named with letters from the alphabet (e.g., A, B, etc.).

Before the experiment starts, participants are requested to choose one of 5 types of artificial agents to play the game in their place (see Fig. 5c). Thus, in this treatment, participants make a single decision. For convenience, we attribute names to the available agent, i.e., always-0, always-2, always-4, compensator and reciprocal. However, during the experiment, agents were only presented by an alphabet letter (e.g., A, B, etc.) and there was never any framing related to the fairness of the agent’s behaviours. Afterwards, they are able to observe the decisions of their agent and the other agents in the group throughout the game. They can also observe the content of the public and private accounts. At each round, participants have a maximum of 10 seconds to observe the information in the screen. Once the game ends, the result is shown on the screen, including information about the participant’s final payoff. Finally, participants are requested to complete a short questionnaire that helps elucidate their decision to select a particular agent and how they experienced the performance of their agent.

### Description of the agents

The first three agents (*always-0*, *always-2*, *always-4*) display unconditional behaviours, i.e., they contribute in every round 0, 2, or 4 EMUs respectively. The last two exhibit conditional behaviours, which respond to external stimuli, in this case, the total contributions of the group members in the previous round ($a_{-i}(t-1)$), in function of a personal threshold $\tau _{p}$, which defines a trigger for behavioural change in the autonomous agents. Agents compare external stimuli to this threshold and adapt their contribution levels depending on the comparison result.

In this treatment, we impose $\tau _{p}=10$ EMUs, representing the total amount of contributions in one round made by the other participants. We call this a locally fair amount since if the other members of the group contribute this amount in every round and the focal player also contributes 2 in every round then the threshold of 120 EMUs would be reached by the end of the game and everyone will have the same gains to take home. Given this threshold $\tau _p$, *Compensators* only contribute if $a_{-i}(t-1) \le \tau _{p}$. In contrast, *reciprocal* agents only contribute if $a_{-i}(t-1) \ge \tau _{p}$. All autonomous agents stop donating once the collected contributions reach (or surpass) the collective target. These agent types emulate the behaviors identified in the *human* control treatment^19^.

### *Customize* treatment (T2)

In the customization treatment, participants are requested to configure an artificial agent that will act in their place during the experiment. Again, in this treatment, participants make a single decision in the experiment. Each artificial agent can be customized in such a way that it can adapt its action to the actions of the other agents within the group, i.e., participants can make a conditional agent. To do this, participants are requested to configure the values of 5 parameters (see Fig. 5b) that define an agent’s behaviour:

- Threshold ($\tau _{p} \in [0..20] \text { in steps of 2}$): is an integer that will be compared to the total contribution of the other group members in the previous round, i.e., the agent will select one of the three following actions based on whether the contributions of the other members of the group in the previous round ($a_{-i}(t-1)$) are greater, equal or lesser than the threshold.
- Initial action ($a_{0}\in {0,2,4}$): the action the agent will take in the first round of the game.
- Action above ($a_{a}\in {0,2,4}$): the action the agent will take if $a_{-i}(t-1)>\tau _{p}$.
- Action equal ($a_{m}\in {0,2,4}$): the action the agent will take if $a_{-i}(t-1)=\tau _{p}$.
- Action below ($a_{b}\in {0,2,4}$)): the action the agent will take if $a_{-i}(t-1)<\tau _{p}$.

Allowing participants to configure their agent effectively increases the behavioural space that can be displayed in the CRD in comparison to the delegate treatment. Concretely, there is a maximum of $10*3^{4}=810$ possible behavioural combinations in which an agent can be configured. Thus, if we observe an increase in collective success in both treatments, we can rule out that this result is caused by a limited number of choices, and instead we may conclude that it is an effect of delegating to autonomous agents. At the same time, we can still classify these behaviors (as was done for Fig. 2b) into roughly 4 categories (see Fig. 5a): cooperate and defect (which are unconditional strategies); and reciprocate and compensate (conditional ones). Finally, in order to assess the level of trust in the delegation system, we ask participants once the experiment has finished, whether, if given the option in another experiment, they would decide to delegate/customize again, or play the game themselves.

### *Nudge* treatment (T3)

In the hybrid experiment, we performed the same setup as described in the human control treatment. Except that in this case, each group is formed by 3 human participants and 3 artificial agents. Participants are told that the agents have been designed to adopt a human-like behaviour in the CRD experiment: we selected the agents’ behaviours from the pool of agents that were part of successful groups in the customize treatment. Nevertheless, participants were only told that that the “artificial agents were designed by humans to act on their behalf in the context of this experiment” (see instructions in the SI). Since we select only agents programmed by participants from successful groups (of the customize treatment), our hypothesis was that those agents may be able to *nudge* human participants into cooperative behaviors.

At the end of the experiment, we test whether participants were able to discern which members of the group were human and which were not. The participants are not informed who the agents are in the session in order to ensure that they are just responding to the actions and not the fact that the action is performed by a human or an agent.

### Sample and collected data

A total of 246 participants were part of this experiment, of which 174 participated in the 3 treatments that involve autonomous agents. The experiment was carried out at the Vrije Universiteit Brussels. The population was mainly composed of university students between the ages of 19 and 33, of which $49.4\%$ were female. The *delegate* treatment had $n=90$ participants divided into 15 groups of 6 participants each. The *customize* treatment had $n=48$ participants and 8 groups. Finally, the *nudge* treatment had a total of $n=48$ participants and 16 hybrid groups. Moreover, the *human* control treatment was part of another experiment^19^ and consisted of $n=72$ participants divided into 12 groups of 6 participants. Due to the COVID-19 situation, we were unable to perform more sessions of the *customize* and *nudge*. Nevertheless, our current data already hints towards interesting phenomena.

For all treatments, we stored the result of the experiment, the configuration over all rounds and the response to a final questionnaire. In the *delegate* (and *customize*) treatment we also stored, for each participant, the selected (customized) agent and the time taken to select it. For the *nudge* treatment, we collected the contribution at each round, the actions of the other members of the group in the previous round, the content of the private and public accounts at each round, the predictions of the total content of the public account after each round, and the time taken to make each decision. We also stored the responses to a follow-up survey that participants had to complete once the experiment was finished. In this regard it is important to mention that 1 participant has been excluded from the survey results in the delegate treatment, since the participant did not complete the survey.

### Ethics declarations

The experiments presented in this manuscript have been reviewed and approved by the Ethical Commission of the Vrije Universiteit Brussels. All experiments were performed in accordance with the Europenean Union GDPR guidelines and regulations, and the study was conducted in accordance with the Declaration of Helsinki. We obtained informed consent from all participants in this experiment. All the data of the experiment has been anonymized and cannot be linked to any participant.

## Supplementary Information

Supplementary Information.

## Data Availability

All the data used in this manuscript has been submitted to Datadryad and can be accessed at 10.5061/dryad.0rxwdbs1z.
