# Supplementary material for: Delegation to artificial agents fosters prosocial behaviors in the collective risk dilemma
Source: Sci Rep. 2022 May 19;12:8492. doi: 10.1038/s41598-022-11518-9 (PMC9119388; doi:10.1038/s41598-022-11518-9)
Supplement: Supplementary file 1 — Supplementary Information. [file 41598_2022_11518_MOESM1_ESM.pdf]

Supplementary Information - Delegation to artificial agents fosters prosocial behaviors in the collective risk dilemma

Elias Fernández Domingos, Inês Terrucha, Rémi Suchon, Jelena Grujić, Juan C. Burguillo, Francisco C. Santos, Tom Lenaerts

Supplementary Figures

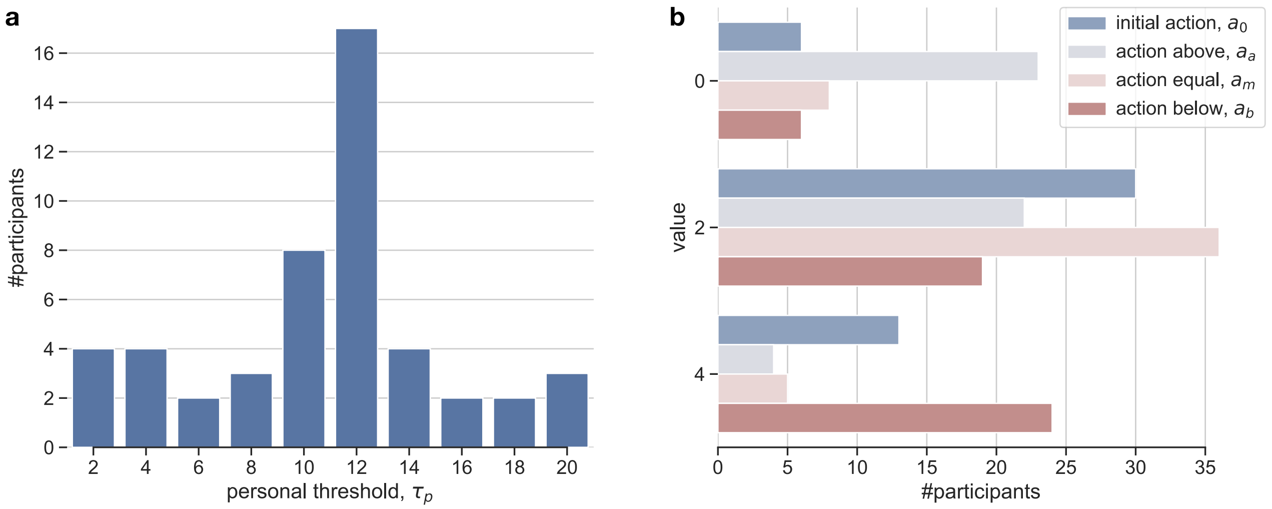

Supplementary Figure S1. Distribution of values of each configurable parameter. Panel (a) shows the distribution of personal thresholds, which displays a preference for $\tau_p = 12$. Panel (b) displays the distribution of the configurable actions. Action 4 was the most frequent choice for parameter a_b and action 0 the most frequent for parameter a_a. These results highlight a preference for compensatory behaviours.

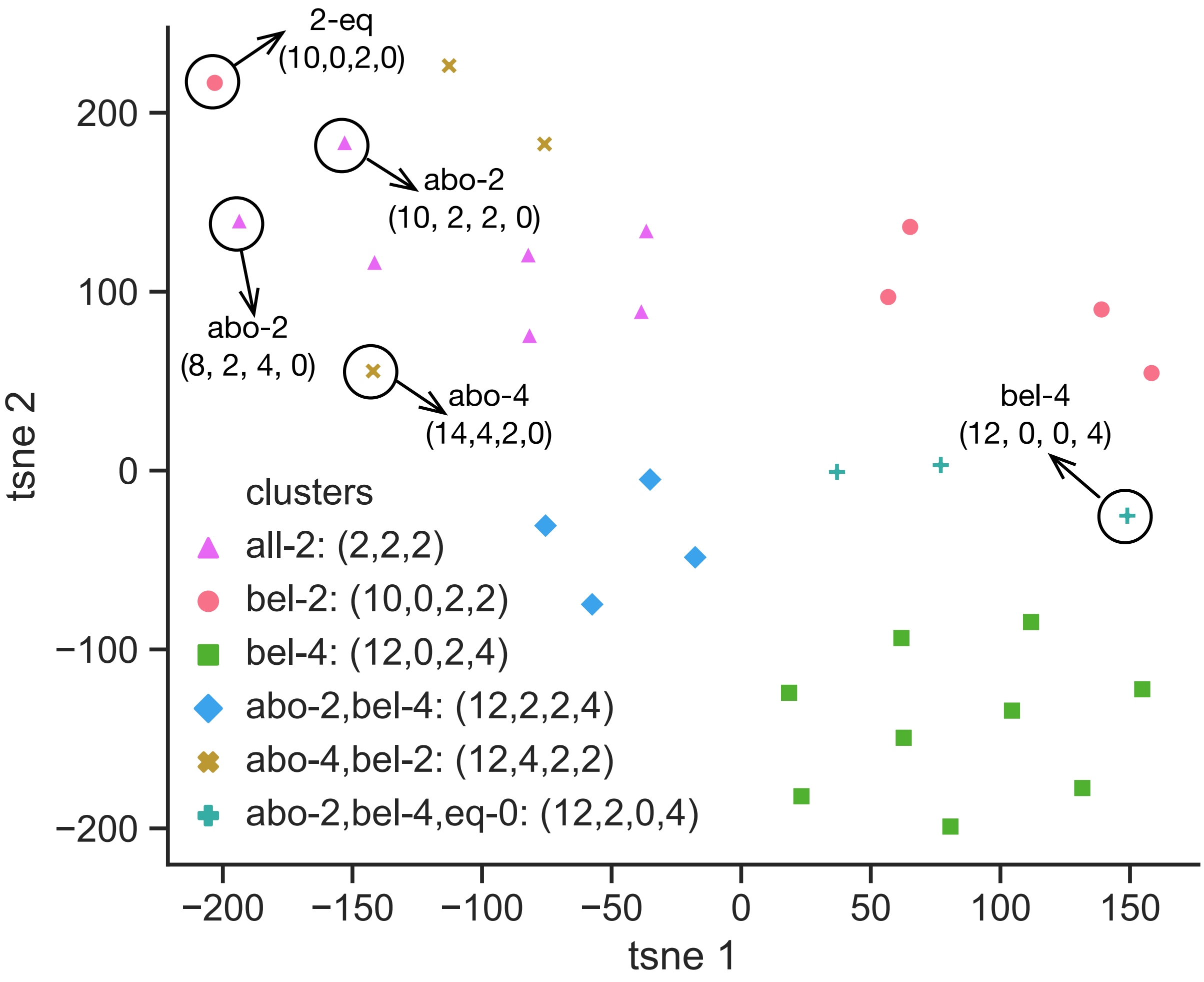

Supplementary Figure S2. Distribution of agent configurations in the customize treatment. Panel (a) shows a 2-dimensional t-SNE representation of the agent configurations in the intermediary levels of τ_p. The coloring into 6 groups is done through a k-means clustering, which issues a few outliers that are indicated in the figure.

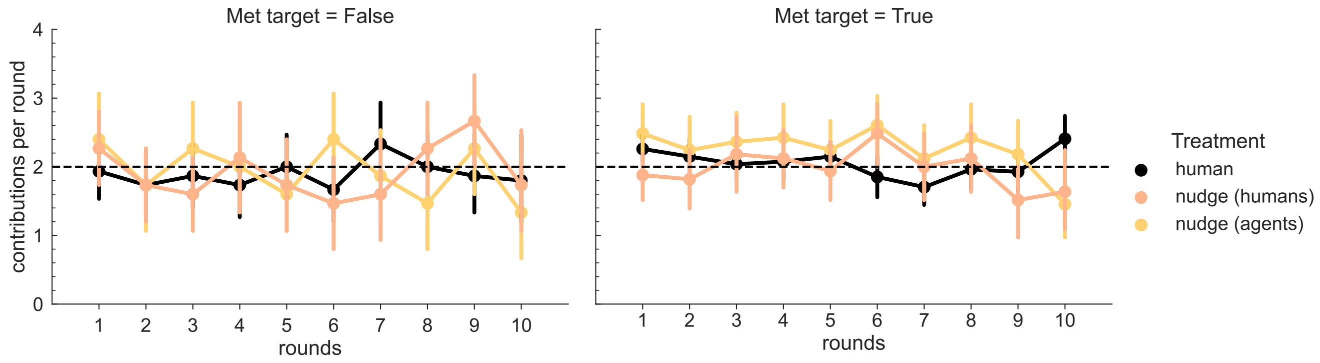

Supplementary Figure S3. Average individual contributions per round. The left panel shows the average individual contributions per round in the NU ($n = 240$) and nudge ($n = 300$) in total for humans and agents) treatments for failed groups, and the right panel shows the same for successful groups ($n = 480$). The contributions of agents ($n = 330$) and humans ($n = 330$) are separated for the nudge treatment.

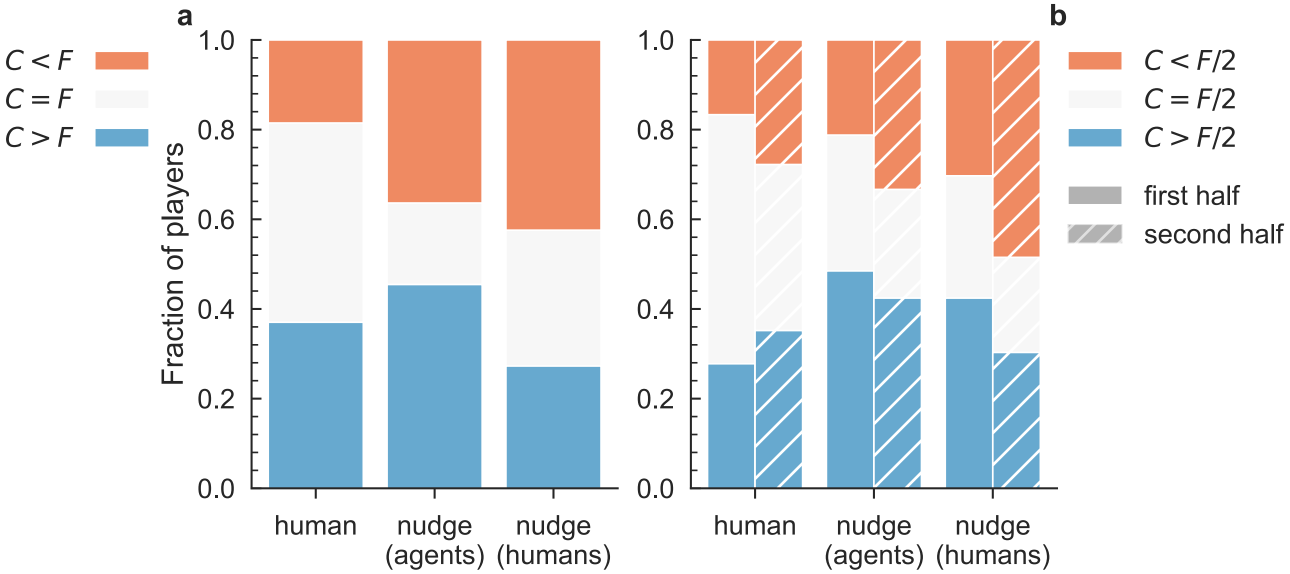

Supplementary Figure S4. Polarization of contributions in the *nudge* treatment in successful groups. Panel (a) shows that the contributions of humans and agents in the *nudge* treatment is more polarized, with most participants contributions either $C > F$ or $C < F$, where F represents half of the endowment and is considered the fair contribution in this experiment. Panel (b) shows that differently from the *humans* treatment, both agents and humans in the *nudge* treatment contribute $C > F/2$ in the first 5 rounds of the game, indicating that the earlier contributions increase, which is important to avoid coordination problems in the last rounds of the game.

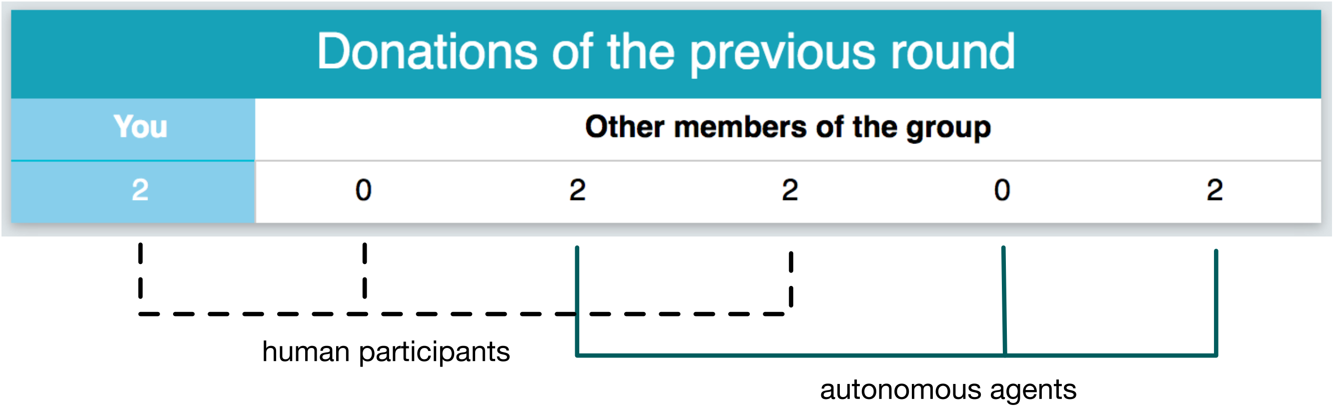

Supplementary Figure S5. Order in which the members of a group are shown during each round of the *nudge* experiment. In each round of the *nudge* experiment, participants can see how much they contributed in the previous round (member 1) and how much the other members of the group contributed in the previous round. Although all participants are anonymous, members of the same group are shown always in the same order, so that participants can form a history of decisions. Members 1 (the focal participant), 2 and 4 are always human, and members 3, 5 and 6 are always artificial agents. Use fix this order, so that we can test at the end of the experiment whether participants are able to identify the artificial agents (see Figure S6).

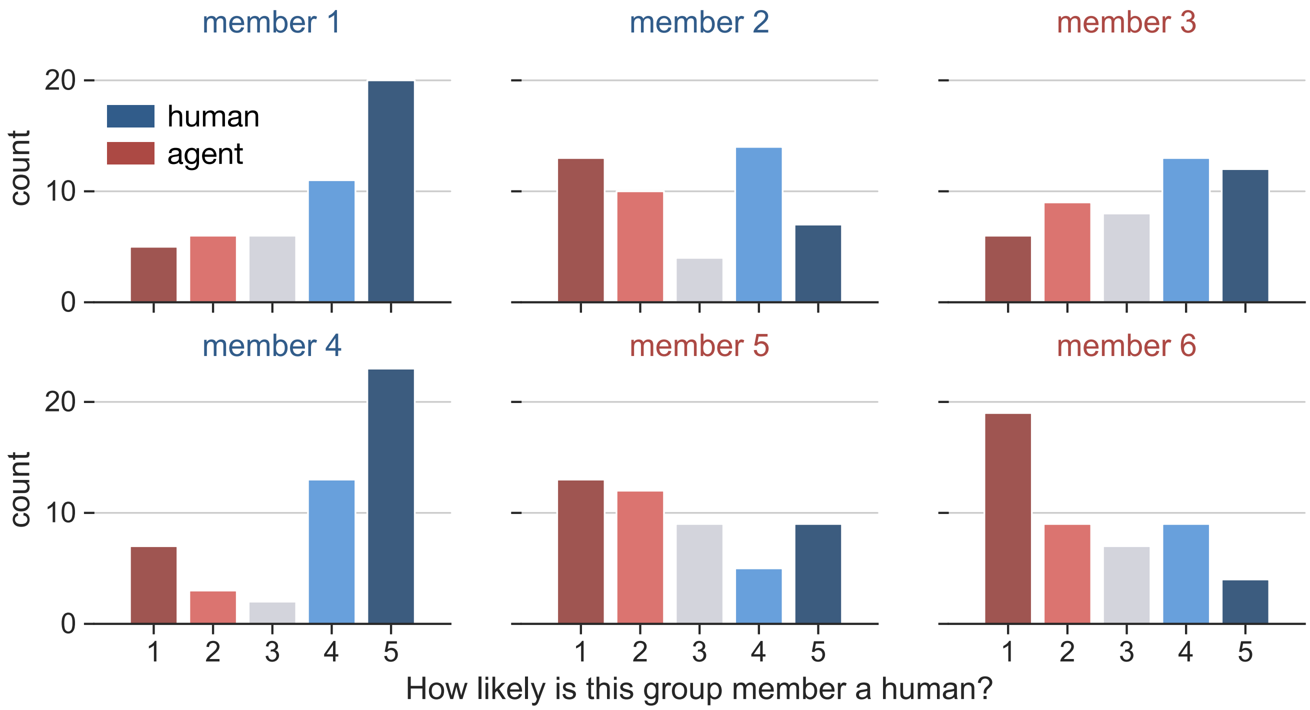

Supplementary Figure S6. Results of the question about the likelihood of a group-member being a human or an agent. Each subplot shows in a scale from 1-5 how likely the group member $i \in [1,6]$ is a human. A 5 means that the participants is sure that the member is a human, while a 0 means that the participant is sure that the member is an artificial agent. A 3 means that the participants is unsure. The order is related to how participants are displayed during the game (see Figure S5). Members 1, 2 and 4 are always human participants and members 3, 5 and 6 are always agents. The results indicate that participants understand that member 1 and 4 are humans and that member 6 is an agent, but they are very unsure about all the other participants.

Experimental Instructions

Instructions for the control treatment (human)

Each participant had access to the following instructions, both in digital and paper format:

Instructions to the experiment

Welcome to this experiment where you can earn money!

You are about to participate in an experiment on iterative decision-making, conducted by researchers from the *Vrije Universiteit Brussel* and the *Université Libre de Bruxelles*. In this experiment, you will earn some money, and the amount will be determined by your choices and the choices of the other participants.

Your privacy is guaranteed: The other participants will not know who you are during the experiment and the results of the experiment are stored in an anonymous manner.

It is very important that you remain silent during the whole experiment, and that you never communicate with other participants, neither verbally, nor in any other way. When in doubt or when you have a question, please just raise your hand and an experimenter will approach you. If you do not remain silent, or if you behave in any way that could potentially disturb the experiment, you will be asked to leave the laboratory, and you will not be paid.

All your earnings during the experiment will be expressed in Experimental Monetary Units (EMUs), which will be transformed into Euros with a change rate of 0.75 Euro to 1 EMU. At the end of the experiment, a show up fee of 2.5 euros will be added to your earnings.

You will be paid privately by bank transfer to your account within a week after the experiment. At the end of the experiment you will be requested to provide your **IBAN number and BIC code** to make the transfer.

Before starting, you will be randomly assigned into a group. You will never know the identity of the other participants of the group. However, the experiment takes 10 rounds and you will be able to observe the actions of the previous round of every member of your group, starting from round 2.

Login to the experiment

Before the experiment can start, please, enter the user login and password you have been given into the login page displayed in the browser of the computer assigned to you.

Once you have logged in, you will be able to see on your screen the same instructions that are written on this paper.

Wait for the instructor's signal before you proceed.

General Information

At the beginning of the experiment you will be randomly assigned to a group, which will include 5 other randomly selected participants.

During the whole experiment, you will interact only with those 5 other group members.

At the beginning of the experiment you and each other group member will receive **a personal endowment of 40 EMUs**.

The whole experiment consists of 10 rounds of the following game.

In each round of the game, you have to decide whether to add 0, 2 or 4 EMUs in a **public account**.

If the public account contains at least **120 EMUs** after the 10th round, **each member of your group will keep their savings**, i.e. the EMUs of your endowment that were **not** put in the public account.

However, **if this minimum is not reached**, the computer will "throw a virtual dice" and **each group member will lose his or her remaining EMUs with a 90% chance (9 times out of 10)**.

Thus, with a 10% chance (1 out of 10) you will keep the remaining EMUs in your private account.

Course of Action

Every round has the same structure and consists of the following **steps**:

Step 1: Choice of how much to contribute (0,2 or 4).

Step 2: Make a prediction about the amount in the public account.

When the experiment reaches its final round, you will move to the final 3rd step:

Step 3: Check if the threshold of the public account has been achieved and calculation of final payoffs

Step 1: The contribution choice

In the Step 1, every member will be asked **"How many EMUs do you want to contribute to the public account"**. Three buttons are provided: **0, 2 and 4 EMUs**. You can select the amount by clicking the button, as is shown in the figure below:

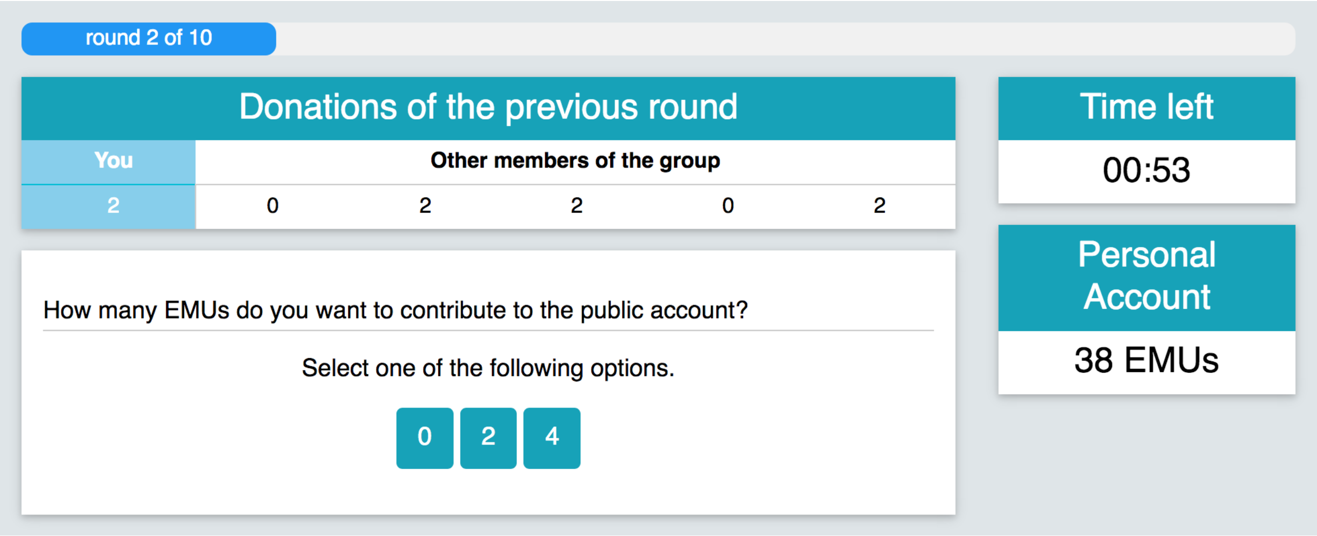

Supplementary Figure S7. View of Step 1.

On the right side of the screen you can see the time you have left to make your decision and the amount of EMUs in your **"Personal Account"**. **You must make your decision within the time displayed on the screen**. The "Time left" square will start blinking when you are getting out of time. Nothing happens when the time runs out, yet if you take too long to make a decision the experiment will take too long. Please respond as quickly as possible.

The table "Donations of the previous round" shows the values donated by all the members of **your group** in the previous round. In the first column, you see **your own donation** from the previous round. In the other columns, you see the decisions of the other users. The choice of each group member will always be shown in the same column. This information about the previous donations is only available after the first round.

Step 2: Predict the content of the public account

After step 1, you will go to a next screen. On this screen, you are asked the following question: **"Please, estimate the current total content of the public account"**. You should enter an estimation of how many EMUs you think the public account contains in total after all members (including you) have made their donations in the current round.

This is an example of what you will see in this step:

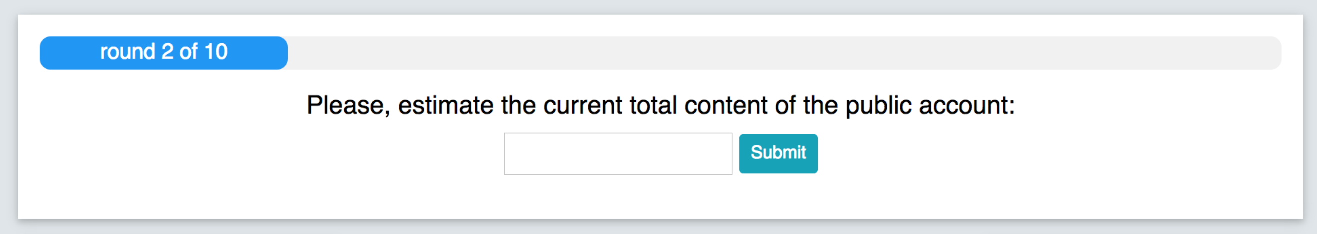

Supplementary Figure S8. View of Step 2.

Step 3: Last round and calculation of final payoffs

After the last round, you will jump to a final screen.

If the accumulated contributions to the account are equal or higher than **120** EMUs, then you will be informed that you can keep the amount of the endowment that you **did not** put in the public account.

For example, if you put in total 20 EMUs of your endowment (40 EMUs) in the public account during the experiment, you will gain the remaining **20** EMUs (i.e. 40 - 20). This amount is converted into Euro's.

The screen will show the following text: "*CONGRATULATIONS! Your group collected XXX EMUs, which is greater or equal to 120 EMUs. So* ***you may keep the amount remaining in your private account****. Please fill in the amount in Euro's you see on this screen on the payment document you received before clicking the continue button. This amount consists of both your private winnings and the show-up fee.*"

However, **if the minimum of 120 EMUs is not reached**, the computer will "throw a virtual dice" and **all group members will lose all their remaining EMUs with a 90% chance**. There are thus two possible outcomes:

On one hand, **with a chance of 9 out of 10, the screen will show**: "*Your group collected XXX EMUs, which is lower than 120 EMUs. The server has generated a random number between 1 and 100. The resulting value is YYY, which is smaller than 91. This means that you all* ***lose the remaining endowment*** *in your private accounts. Please fill in the amount in Euro's you see on this screen on the payment document you received before clicking the continue button. This amount is the show-up fee.*"

On the other hand, **with a chance of 1 out of 10, the screen will show**: "*Your group collected XXX EMUs, which is lower than 120 EMUs. The server has generated a random number between 1 and 100. The resulting value is YYY, which is bigger than 90. This means that you all* ***win the remaining endowment*** *in your private accounts. Please fill in the amount in Euro's you see on this screen on the payment document you received before clicking the continue button. This amount consists of both your private winnings and the show-up fee.*"

End of experiment questionnaire

At the end of the experiment you will be directed to a form containing a short questionnaire. Please answer to all the questions honestly, the information you add here is an important part of this experiment. Any information that you may include in this form will remain completely anonymous and cannot be linked to you in any way. Once you have finished filling in the questionnaire, please, click the button **submit**.

At the end of the experiment, you will be called by one of the organisers to make the payment official. Please stay seated and do not talk until you are called and have left the room.

Please note:

Communication is not allowed during the whole experiment. If you have a question, please raise your hand.

All decisions are made anonymously, i.e. no other participant learns the identity of the other decision makers.

The payment is also anonymous, no participant learns from us about the amount that another participant received in the

experiment.

Delegate treatment

The instructions of delegate treatment differ from the *human* control in that participants do not make their own contributions to the public account during the experiment, but they have to select an autonomous agent that will make them in their place. Below we describe only the sections that change with respect to the control treatment (NU).

General Information

At the beginning of the experiment, you will be randomly assigned to a group, which will include 5 other randomly selected participants (in total, each group will have 6 participants).

During the whole experiment, you will interact only with those 5 other group members.

At the beginning of the experiment, you and each other group member will receive **a personal endowment of 40 EMUs**, which will be stored in your **private account**.

Afterwards, each participant will be requested to choose one of **5 possible artificial agents**, whose behaviour will be described in detail. The agent will be in charge of choosing how much to invest from the **private account** of the participant to the **public account** over the **10 rounds** of the experiment.

In each round of the game, the artificial agent will have to decide whether to add 0, 2 or 4 EMU in a **public account**. You will be able to observe the actions of the agent and of the other agents in the group, the content of the **public account** and the content of the **private account** where the remainder of your **endowment** is stored.

If the public account contains at least **120 EMUs** after the final round, **each member of your group will keep their savings**, i.e. the EMUs of your endowment that were not put in the public account.

However, **if this minimum is not reached**, the computer will throw again "a virtual dice" and **each group member will lose his or her remaining EMUs with a 90% chance (9 times out of 10)**.

Thus, with a 10% chance (1 out of 10), you will keep the remaining EMUs in your private account.

Course of Action

At the beginning of the game, each participant **must** choose an artificial agent that will act in her/his place during the experiment:

Step 1: Choice of which artificial agent to perform the experiment.

Afterwards, every round has the same structure and consists of the following **step**:

Step 2: The artificial agent chooses of how much to contribute (0,2 or 4 EMUs).

When the experiment reaches its final round, you will move to the final 3rd step:

Step 3: Check if the threshold of the public account has been achieved and calculation of final payoffs

Step 1: Select an artificial agent

In Step 1, every member of the group will be asked: "**Choose an artificial agent to perform the experiment in your place**".

There is a **"virtual fair dice"** for each group. Thus, the experiment can have different rounds, depending on which group you are in.

You will be able to choose among **5** possible artificial agents. You will be able to observe a **detailed description of the behaviour of each agent on your screen**.

Once you have chosen an agent, **it will act in your place during the rest of the experiment**, and you will not be able to intervene anymore in its outcome. However, you will be able to observe the actions of the agent and the rest of the agents

in the group during each round, as described in Step 2. All artificial agents will stop contributing money from your **private account** (will start choosing action 0 EMUs) once the target has been reached (the content of the **public account is bigger or equal to 120 EMUs**).

Here is a description of all the artificial agents you may select (you can observe the same information in your screen during the experiment):

Agent A: This agent **contributes 0 EMUs on every round**.

Agent B:

1. This agent **always contributes 2 EMUs** in the 1st round. In the following rounds it will:
2. **Contribute 0 EMUs** if the other agents in the group contributed, in total, less than (<) 10 EMUs in the previous round. (This means that the other agents in the group contributed on average less than 2 EMUs on the previous round).
3. Otherwise, it will **contribute 4 EMUs**.
4. Once the public account contains at least 120 EMUs, it will contribute 0 EMUs.

Agent C:

1. This agent **contributes 2 EMUs** on every round.
2. Once the public account contains at least 120 EMUs, it will contribute 0 EMUs.

Agent D:

1. This agent **always contributes 2 EMUs** in the 1st round. In the following rounds it will:
2. **Contribute 4 EMUs** if the other agents in the group contributed, in total, less than or equal to ($\geq$) 10 EMUs in the previous round. (This means that the other agents in the group contributed on average less than or equal to 2 EMUs on the previous round).
3. Otherwise, it will **contribute 0 EMUs**.
4. Once the public account contains at least 120 EMUs, it will contribute 0 EMUs.

Agent E:

1. This agent **contributes 4 EMUs** on every round.
2. Once the public account contains at least 120 EMUs, it will contribute 0 EMUs.

Step 2: The contribution choice

In Step 2, every agent will choose how much to **contribute to the public account** among one of the following 3 choices: **0, 2 and 4 EMUs**. This is an example of the screen you will be able to observe at each round:

You will have **10 seconds** to observe the information on this screen before being redirected to the next round. You may also press “continue” before this time frame, if you are ready.

On the right side of the screen you can see the time you have left to observe the screen, the amount of EMUs in your private account, and the total amount invested in the public account up until the current round.

The table “Donations in the previous round” shows the values donated by all the artificial agents of **your group** in the previous round. In the first column, you see the donations of your agent from the previous round. In the other columns, you see the decisions of the other agents. The choice of each artificial agent will always be shown in the same column. This information about the previous donations is only available after the first round.

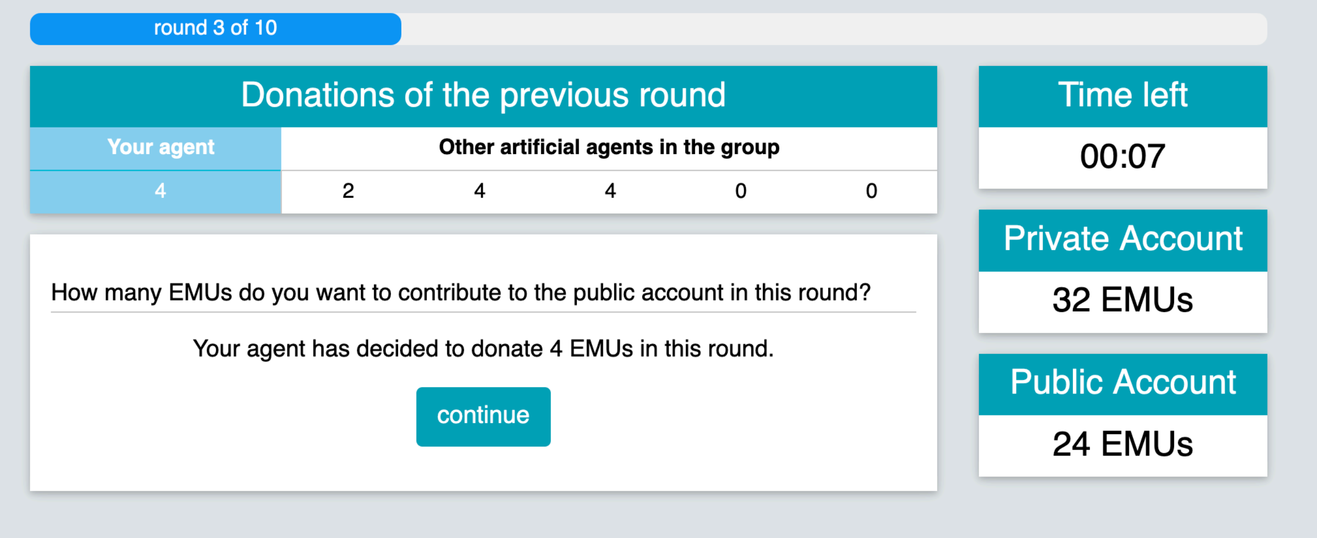

Supplementary Figure S9. View of Step 2 for the delegate treatment.

Step 3: Last round and calculation of final payoffs

After the last round, you will jump to a final screen.

If the accumulated contributions to the account are equal or higher than **120 EMUs**, then you will be informed that you can keep the amount of the endowment that your agent **did not** put in the public account.

For example, if your agent put in total 20 EMUs of your endowment (40 EMUs) in the public account during the experiment, you will gain the remaining 20 EMUs (i.e., 40 - 20). This amount is then converted into Euros.

The screen will show the following text: *"CONGRATULATIONS! The artificial agents in your group collected XXX EMUs, which is greater or equal to 120 EMUs. So* ***you may keep the amount remaining in your private account****. Please fill in the amount in Euro's you see on this screen on the payment document you received before clicking the continue button. This amount consists of both your private winnings and the show-up fee."*

However, **if the minimum of 120 EMUs is not reached**, the computer will "throw a virtual dice", and **all group members will lose all their remaining EMUs with a 90% chance**. There are thus two possible outcomes:

On the one hand, **with a chance of 9 out of 10, the screen will show**: *"The artificial agents in your group collected XXX EMUs, which is lower than 120 EMUs. The server has generated a random number between 1 and 100. The resulting value is YYY, which is smaller than 91. This means that all the group members will* ***lose the remaining endowment in their private accounts****. Please fill in the amount in Euro's you see on this screen on the payment document you received before clicking the continue button. This amount is the show-up fee."*

On the other hand, **with a chance of 1 out of 10, the screen will show**: *"The artificial agents in your group collected XXX EMUs, which is lower than 120 EMUs. The server has generated a random number between 1 and 100. The resulting value is YYY, which is bigger than 90. This means that all the group members will* ***win the remaining endowment in their private accounts****. Please fill in the amount in Euros you see on this screen on the payment document you received before clicking the continue button. This amount consists of both your private winnings and the show-up fee."*

Post-experiment questionnaire

At the end of the experiment, each participant has to complete a short survey. We designed the survey to provide a deeper knowledge about the delegation choices of each participant and their motivations. Here are some of the questions we have asked:

- If the experiment was repeated, would you select the same agent? If not, select the option with the agent you would choose: i) agent A, ii) agent B, iii) agent C, iv) agent D, v) agent E. If you had the option, would you prefer to play

yourself or choose one of the previous artificial agents?

- If you could choose an agent built with the latest artificial intelligence technology, would you prefer to: i) play yourself, ii) choose an agent that only tries to maximise your personal benefit, iii) choose an agent that tries to maximise the chances to reach the collective target (120 EMUs).
- Which of the following options was the most important for you in your choice of artificial agent? i) fairness, ii) your own profit, iii) maximise the chances of achieving the target, iv) maximise the profit of the group, v) avoid investing more than the other members of the group.

customize treatment

In the customize treatment, participants configure the agent to which they will delegate their actions during the experiment. Here we describe the sections of the instructions that differ from the *delegate* experiment.

General Information

The experiment lasts 10 rounds, and, before round 1, each participant must configure **an artificial agent that will act on her/his behalf during the experiment**, according to a procedure described later.

Course of Action

Before the experiment starts, each participant **must** configure an artificial agent that will act in her/his behalf during the experiment:

Step 1: You configure your artificial agent.

Step 1: Select an artificial agent

Every member of the group has to configure his/her own artificial agent. This artificial agent will make contributions from its owner's **private account** to the group's **public account**.

You configure the artificial agent by setting one **threshold** and four levels of **contribution**.

The levels of contribution define the contribution that the agent will make from your **private account** to the **public account**, in different situations of the game.

First, you will have to configure the contribution the agent will make in **round 1**.

From **round 2 onwards**, your agent will look at the **aggregate contributions of the other members of your group (without you) in the previous round**, i.e., it will look at the sum of contributions of your groupmates. Thus, you will have to configure a **threshold**. **In each round, the aggregate contribution of your groupmates will be compared to this threshold**. Please note that, since the maximum contribution per round is 4 EMUs, the maximum aggregate contributions of your groupmates in any round is 20 EMUs. You will have to configure **three levels of contributions** that your artificial agent will use to decide which contribution to make if the aggregate contributions of your groupmates are higher, equal or lower than the threshold that you have configured.

You may find below a summary description of the configurable parameters of your artificial agent:

Threshold:

A threshold is any even number between zero and twenty: 0, 2, 4, 6, 8, 10, 12, 14, 16, 18 or 20.

Contribution levels:

- Contribution for the first round: 0, 2 or 4 EMUs.
- Contribution if the aggregate contribution of the other group members was below the threshold in the previous round: 0, 2 or 4 EMUs.
- Contribution if the aggregate contribution of the other group members was above the threshold in the previous round: 0, 2 or 4 EMUs.

- Contribution if the aggregate contribution of the other group members was equal to the threshold in the previous round: 0, 2 or 4 EMUs.

Post-experiment questionnaire
In the customize treatment we maintain a very similar final questionnaire to the delegate treatment, with some logical changes:

- If the experiment was repeated would you configure the agent in the same way? If not, indicate which configuration would you choose. Write separated by a comma (,), and in the following order, the threshold, initial contribution, contribution if the aggregate (sum of) contributions of the group mates are above the threshold, if they are equal, or if they are below.
- If you had the option, would you prefer to make your own contributions throughout the experiment or configure an agent to contribute on your behalf?
- Which of the following options was the most important for you when configuring your artificial agent? i) fairness, ii) your own profit, iii) maximise the chances of achieving the target, iv) maximise the profit of the group, v) avoid investing more than the other members of the group.

Nudge treatment

The nudge treatment differs from NU in that half of the participants of each group (of 6) are artificial agents. These agents have been selected randomly from the pool of customized agents of successful groups in the *customize* treatment. Here we indicate only the parts of the instructions that differ from NU.

General Information
In each group, there will be 3 randomly selected **human participants** (including you) and 3 **artificial agents**. The artificial agents were designed by Humans to act on their behalf in the context of this experiment.

During the experiment, you will not know which members of your group are agents and which ones are humans, except for yourself.

Post-experiment questionnaire
In the hybrid experiment we included some extra questions in the final questionnaire to delve into the motivation of participants and better understand how they regarded the hybrid interactions:

- Did the presence of artificial agents influence your actions during the experiment? If yes, how?
- Who do you think contributed more to the public account, agents or humans?
- Do you think that having agents in the group was beneficial for the outcome of the experiment?
- Which of the following options was the most important for you in your choice of contributions during the experiment? i) fairness, ii) your own profit, iii) maximise the chances of achieving the target, iv) maximise the profit of the group, v) avoid investing more than the other members of the group.
- Below you can see the contributions of all the members of your group (including you) during the experiment. Each row corresponds to a different participant/artificial agent and each column to a round. Please indicate how certain you are that these contributions come from a human participant, by answering the following 6 questions (the question below is repeated for each group member):
  - What do you think about member 1? 1. certain the contributions are from an artificial agent; 2. slightly certain the contributions are from an artificial agent; 3. uncertain whether the contributions are from a human or an artificial agent; 4. slightly certain the contributions are from a human; 5. certain the contributions are from a human.
